# The Relationship of School Participation with Motor Proficiency and Executive Function in Children with Autism Spectrum Disorder

**DOI:** 10.22037/ijcn.v15i2.19721

**Published:** 2021

**Authors:** Mahsa KHEIROLLAHZADEH, Mehdi ALIZADEH ZAREI, Malek AMINI, Faezeh DEHGHAN

**Affiliations:** 1 Department Of Occupational Therapy, School Of Rehabilitation Sciences, Iran University Of Medical Sciences, Tehran, Iran.; 2 Department Of Neuroscience, School of New Technologies, Iran University Of Medical Sciences, Tehran, Iran.

**Keywords:** Autism spectrum disorders, Patient Participation, Motor skills, Executive function

## Abstract

**Objectives:**

Participation in meaningful activities is an important aspect of development in children with developmental disorders such as autism spectrum disorder (ASD). The purpose of this study was to assess the correlation of school participation with motor proficiency and executive function in children with ASD.

**Materials & Methods:**

In this cross-sectional (descriptive-analytic) study, 52 students aged 6 to 12 years old with ASD were selected through the convenience sampling method. The GARS-2 scale was used to confirm ASD diagnosis. Other psychiatric comorbidities such as ADHA were studied by the CSI-4 tool, and students with comorbidities were excluded. Data were collected using SFA, BOTMP-2, and BRIEF questionnaires. It should be noted that in the BRIEF questionnaire, a higher score indicates a more severe disability.

**Results:**

Our findings showed that motor proficiency and its components had a significant direct correlation with school participation in children with ASD (P ≤0.001). On the other hand, school participation was inversely and significantly correlated with the behavioral regulation and metacognition monitoring indices of the executive function dimension (P <0.05).

**Conclusion:**

Based on the findings of this research, the development of motor proficiency and improvements in the behavioral regulation and metacognition monitoring of students with ASD will boost their participation in school activities. Motor proficiency was significantly correlated with school participation in children with ASD. More attention should be paid to perceptual motor interventions and cognitive rehabilitation programs (with a focus on monitoring metacognition and shifting behavioral regulation) to increase the participation of children with ASD in school activities.

## Introduction

Participation is a frequently-used concept in occupational therapy and other health sciences. The International Classification of Function, Disability, and Health (ICF) and the Model of Human Occupation (MOHO) have defined and elaborated the concept of participation. According to the MOHO model, participation is defined as engagement with work, play, and the activities of daily living (ADL) in a socio-cultural context, which is essential to give a person a sense of well-being. Similarly, the ICF describes participation as involvement in life situations (1). Participation in meaningful activities is deemed as one of the significant factors boosting the development of children with disabilities. By being engaged in various activities, children acquire new skills, enabling them to achieve competencies and satisfaction (2). Among the major problems of children with autism spectrum disorder (ASD) are their limitations in participating in social and daily activities. The studies conducted on this issue indicate that the diversity of the activities performed by these children is less than that of their peers . These limitations have been investigated from various angles such as self-care, community mobility, social communications, play and leisure, education, etc. (3). 

School participation refers to the student’s ability to grasp opportunities and actively participate in the roles corresponding to his/her age and culture (4). Participation in school activities has been noted to enhance ASD children’s well-being and quality of lives (2). The unique characteristics of children with ASD influence their experiences at school (5). Although numerous studies have been conducted on the participation of children with disabilities in school activities, such studies on children with ASD have been neglected despite the increasing number of these children (6). In their research on children with ASD, Vatrayan *et al*. (2015) reported that visual perception and the imitation of movements were associated with the children’s levels of participation in school activities (7). Hsiao *et al*. (2013) declared that autism children’s social characteristics played significant roles in their adaptation to school (8). Ashburner *et al*. (2010) examined children with ASD in terms of school function and emotional and behavioral regulations, and their results showed that compared to their peers, 54% of children with autism had lower levels of school function and greater difficulties in behavioral and emotional regulations and had experienced some problems such as anxiety, depression, attention deficit disorder, opposite behaviors, and aggression (9). In 2008, Ashburner studied the impact of sensory processing on the educational, behavioral, and emotional performance of children with ASD at school. The results revealed that children with sensory-seeking problems and those with difficulty in filtering sounds in crowded environments performed poorer school functions (10). One of the most important factors determining occupational performance is the cognitive ability. The ICF highlights the role of cognition in performing ADLs and incorporates it into the body function classification. Various psychological models of cognitive defects have been proposed in autism, one of which is the executive function theory (6). The executive function is defined as a high-level cognitive ability, which brings about purposeful behaviors in new and challenging situations (11). Pellicano *et al*. (2017) examined the role of executive function in the school readiness of children with ASD. Compared with the control group, ASD children had a poor executive function and were less ready to enter school. Also, the executive function, especially at the working memory and inhibition dimensions, was significantly associated with the readiness of both groups to enter school (12). Sasser *et al*. (2015) observed a significant relationship between the executive function of preschool children and their future school function and social excitement (13). In their research on children with ASD, Zingervich *et al*. (2009) found that executive function had a greater impact on school function than on sensory processing (6). 

Motor deficits are among other secondary symptoms of ASD. Hypotonia, motor dyspraxia, and impaired gross skills and fine motor skills are the most common motor disorders in children with autism (14). Motor skills pertain to adaptive behaviors and daily life, communication, and social skills (15). Since motor disorders affect the functional capacity of children, it is of crucial importance to diagnose and treat these problems at an early age (16). Based on the studies conducted on this issue, motor deficits in preschool children would result in poor school performance (17); nevertheless, early motor interventions have been neglected in preschool programs for autistic children (15). Gates *et al*. examined the relationship between academic performance and motor functions in children with cerebral palsy (CP) and concluded that there was a significant correlation between daily motor functions and academic performance in these children (18). Given the participation problems faced by children with ASD at school and the role of occupational therapists in school-based interventions, identifying the factors associated with school participation in these children seems to be beneficial for implementing effective interventions. Therefore, considering the points raised, the present study was conducted to determine the relationship between school participation and motor proficiency and executive function in children with ASD.

## Materials & Methods

**Procedure**

This was a correlational and cross-sectional (descriptive-analytic) study. Participants included all 6- to 12-year-old students with ASD who were under education in special schools, recruited by the convenient available sampling method. Inclusion criteria were as follows: the diagnosis of ASD by a psychiatrist, the absence of other psychiatric disorders such as the attention-deficit/hyperactivity disorder (based on the GARS-2 test), an age of 6 to 12 years, and attendance at schools for autistic children. 

Iran University of Medical Sciences approved the research and issued an ethical approval certificate (code: IR.IUMS.REC1395.9411255002). Afterward, the Faculty of Rehabilitation Sciences provided the researcher with a referral form to the Special Educational Department (Tehran), which then gave the researcher an introduction letter for referring to the schools of autistic children. Three special schools for children with ASD, located in different districts of Tehran, were selected. The researcher visited these schools and then, with the aid of the school principal, identified and invited the parents of 6- to 12-year-old students to a meeting at school. Over the course of the meeting, the consent form was filled out by volunteered parents, and the CSI-4, GARS-2, and BRIEF questionnaires were given to them. The parents were asked to complete the questionnaires and return them to the school principal within three days. Sixty-seven parents filled out the questionnaires; however, due to the diagnosis of comorbidities, the forms of children with ADHD (n=15) were excluded from the study, and the questionnaires received from the rest of the parents (n=52) were analyzed. Afterward, the School Function Assessment (SFA) questionnaire was administered to the children’s teachers to fill it out. Teachers also responded to a series of questions, for some of which they asked assistance from the school principal, the sports teacher, and caregivers. The BOTMP-2 motor proficiency Test was run in a room at the school. The test’s subcomponents were administered in such an order that was consistent with each student’s condition. In this regard, for the evaluation of the students who had less sustained attention and couldn’t tolerate sitting in their places, the tests started with the subcomponents of gross motor skills and coordination. If the child could tolerate the test on the same day, he/she was asked to keep on performing fine motor skills; otherwise, the rest of the assessment would be postponed to another day. Some students did not cooperate well in performing the tests of gross motor skills and coordination and were more willing to do paper and pencil tasks; so, their assessment began by testing their subtle fine motor skills. Likewise, based on students’ conditions, the tests were either administered on the same day or postponed to the next days.

*Outcome measurements*

School Function Assessment 

School Function Assessment (SFA) was developed by Coster *et al*. in the United States in 1998. The validity and reliability of this test have been confirmed in various countries, including Iran [(19). The Cronbach’s alpha values of this tool have been measured in two studies on 23 and 29 students, reporting the values of 0.92 and 0.98, respectively. The internal consistency values of the tool in these two studies were reported 0.82 and 0.92, respectively. This tool is deemed a comprehensive assessment tool for evaluating the function and participation of children in preschool activities up to the sixth grade. The time required to complete the form varies from 45 minutes to one hour. The SFA includes 320 items categorized into three sections as follows: participation, task support, and activity performance. In the section related to participation, the therapist evaluates students’ participation in the non-academic activities occurring in any typical or specific educational environment (depending on the situation in which the student receives education most of the time). In this part, the levels of students’ participation in six major school settings, including regular or special educational classes, playground or recess, transportation to and from school, toileting activities, transitions to and from classes, mealtime, or snack time, are assessed. Items can be completed by teachers, occupational therapists, speech and language pathologists, physiotherapists, teachers, and other support staff. Each of the aforementioned groups can answer the items relevant to them (4).

**Behavior Rating Inventory of Executive Function**

The Behavior Rating Inventory of Executive Function (BRIEF) is a test that evaluates executive functional defects in people aged from 5 to 18 years old. It consists of 86 items and has two forms, namely the parent- and teacher-report versions. The test consists of two parts, including the behavioral-regulation index and the metacognition index. The time required to complete the form is between 15 and 20 minutes. The Behavioral-regulation index contains three subsets, namely inhibition, shift, and behavioral regulation, and the metacognition index includes five subsets, namely initiation, planning and organization, material organization, monitoring, and working memory. Each item can be scored on a three-point Likert scale, i.e., never (0), rarely (1), and often (2). This test has a high test-retest reliability (ICC=0.88 for the teacher-report version and ICC=0.82 for the parent-report version) (20). In Iran, the validity and reliability of this questionnaire were investigated in a study by Salman *et al*. in 2016, who observed that the internal consistency of all of the items was greater than 0.85, and the test-retest reliability was greater than 0.7, indicating satisfactory reliability (21). 

***Bruininks Oseretsky Test of Motor Proficiency***

The Bruininks Oseretsky Test of Motor Proficiency (BOTMP-2) is a norm-referenced test that evaluates the motor function of children who are at the age range of 4 years and 6 months to 14 years and 6 months. The full version of this test consists of eight parts and 46 items. Its short version contains 14 items originated from its full version and provides a brief overview of general motor function. Bruininks modified this test in 1978 via enriching the Oseretsky Motor Proficiency Test and standardized it based on a sample of 756 children enrolled in the 1970 census in terms of age, gender, race, population size, and geographical areas. The ICC and α coefficients were reported to be 0.87 and 0.84, respectively. The completion of the full and short versions of the tool takes 45 to 60 minutes and 15 to 20 minutes, respectively. Each of the eight parts has been designed to scrutinize a specific aspect of motor development (22).

***Gilliam Autism Rating Scale-2***

The Gilliam Autism Rating Scale-2 (GARS-2) (Gilliam, 1995) is a tool utilized for diagnosing and screening autism in people aged 3 to 22 years. Overall, 1,092 children, adolescents, and adults in the United States and Canada have been screened for autism using this tool. The questionnaire consists of 56 items categorized into four sections (i.e., stereotypical behaviors, communications, social interactions, and developmental disorders). Each item is measured on a four-point Likert scale, i.e., never, rarely, sometimes, and often. The total points are converted to standardized values. The psychometric properties of this tool have been investigated in Iran. Its cut-off point is 52, and the sensitivity and specificity have been 99% and 100%, respectively. Moreover, its reliability has been proven to be high (α= 0.89) (23).

***Child Symptom Inventory-4***

The Child Symptom Inventory-4 (CSI-4) is a behavioral measurement scale that was first developed in 1984 to screen children aged 5 to 12 years for 18 behavioral and emotional disorders. It was revised based on the DSM-IV and released under the name (i.e., CSI-4) in 1994. The questionnaire has two versions, i.e., the parent- and teacher-report versions. The parent-report version has 112 items devoted to 11 major groups of behavioral disorders along with an additional group, and the teacher-report version has 77 items covering nine major groups of behavioral disorders. The internal consistencies (i.e., the α coefficient) obtained for the parent- and teacher-report versions were 0.90 and 0.93, respectively (24).

**Statistical Analysis**

The data were analyzed by SPSS 21 software using the Pearson correlation coefficient. 

## Results

The average age of the children participating in the present study was 117 months. All of the subjects were 6- to 12-year-old boys. Five of them were in pre-elementary, and 25, 8, 5, and 9 were in the first, second, third, and fourth grades, respectively (Table 1). Our results showed statistically significant correlations between school participation and motor proficiency (r = 0.56, p = 0.001) and its components, including gross motor skills (r = 0.44, p = 0.001), motor coordination (r = 0.62, p = 0.00), and fine motor skills (r = 0.56, p=0.00). The total score obtained for executive function (r = -0.18; p = 0.09), the behavioral-regulation index (r = -0.11, p = 0.20), and the metacognition index (r = -0.21, p=0.06) had no significant correlations with participation in school activities. Regarding the subcomponents of the behavioral-regulation and metacognition indices, significant correlations were found between school participation and shift (r = 0.25, p = 0.03) and monitoring (r = -0.29, p = 0.01) (Table 3). It should be noted that in the BRIEF scale, the higher the score is, the weaker the performance will be. So, the observed negative correlation coefficients do not mean that participation has an inverse relationship with the shift and monitoring subcomponents.

## Discussion

This study was designed to assess the relationship of school participation with motor proficiency and executive function in children with ASD. Executive function has two aspects, namely the behavioral-regulation index and the metacognition index. The behavioral-regulation index consists of three subsets, including inhibition, shift, and behavioral regulation, and the metacognitive index consists of five subsets, namely initiation, planning and organization, material organization, monitoring, and working memory. The term "executive function" is an umbrella term covering a set of heterogeneous cognitive functions that interact with each other (25). Therefore, in the present study, besides the overall performance of executive function and its two indices (i.e., behavioral regulation and metacognition), their subsets were also studied in terms of their effects on school participation in children with ASD. The results of this study showed that one subset of the behavioral regulation index (i.e., shift) and one subset of the metacognitive index (i.e., monitoring) had a significant correlation with participation in school activities. The items related to the SFA scale measure the level of participation in six school settings, including playground/recess, transportation to/from the school, toileting activities, transition to/from classes, and mealtime/snack time. The items pertaining to this section engage both behavioral and social, as well as mobility aspects. For example, transportation to the school involves moving from one place or a school’s room to another place or room, which requires queuing, moving in crowded corridors, crossing ports, following commands and rules, and observing social behaviors appropriately. Based on its definition, executive function necessitates a high level of cognitive ability to be activated in new or challenging situations (11). Based on the obtained results, it could be concluded that since school participation encompasses six situations, the observed total scores of the executive function, as well as behavioral-regulation and metacognition subcomponents did not necessarily correlate with the children’s participation in school activities. Regarding the SFA scale, the items related to participation are devoted to the activities that require transition between tasks’ stages or movement between different locations. In their systematic review, Leung and Zakzanis (2014) concluded that children with ASD scored significantly higher in the BRIEF scale, which indicated their weaker performance in transitions between tasks’ stages (26). Due to their weaknesses in cognitive flexibility, children with autism are not able to move to a new location or position (27,28). Therefore, they have difficulty in inward- or outward-moving and in shifts between different stages of a specific task. Another issue related to each of the six subsets of participation is the degree of compliance with rules, regulations, and social behaviors. In their study, Handa and Noro observed a significant relationship between monitoring and social skills (29). In the BRIEF scale, monitoring includes self- and task-monitoring (25). So, it can be concluded that if a student’s ability to monitor his own behavior is weak, his/her participation in school is also weak. In their study, Zingervich and LaVesser (2009) reported that school participation was associated with both behavior-regulation and metacognition indices. The participants in the recent study were the students with ASD attending regular schools (6) while the participants in our study were being educated in special schools for children with autism. Considering this discrepancy, it can be declared that lower disability rates and higher functional rates predict a higher dependency of school participation on the executive function. The findings of this study showed that all of the subcomponents of motor proficiency were significantly correlated with participation in school. In their studies, MacDonald (2013), Scharoun (2015), and Casartelli (2016) emphasized the importance of motor skills in determining social performance and adaptive behaviors in patients with ASD (15,30,31) which was consistent with the findings of the current study. The Bruininks-Oseretsky motor proficiency test consists of three parts, including gross motor skills, motor coordination, and fine motor skills. The items assessing school participation actually scrutinize activities such as moving inside or outside the school, participating in leisure activities in playgrounds, going to the toilet, and eating at meal/snack times, being engaged in which is crucial. Tasks such as participating in classroom activities, eating, and toileting involve the activities requiring working with paper, pencil, and manipulative tools (32-34). Therefore, school participation is the most relevant factor to fine motor skills. On the whole, it can be concluded that the motor proficiency of students with ASD is positively correlated with participation in school activities.

**Table 1:** Patients’ Demographic characteristics

**Cumulative percent**	**Percent**	**Frequency**		
100.0	100.0	52	Male	Gender
9.6	5	5	Preschool	Academic grade
57.7	48.1	25	First grade	
73.1	15.4	8	Second grade	
82.7	9.6	5	Third grade	
100.0	17.3	9	Fourth grade	

**Table 2:** The means and standard deviations of the data in terms of age, school participation, as well as motor proficiency and executive function parameters

**Variables**	**Items**	**Mean**	**SD**
Age (month)		117	17.22
Participation		22.09	7.78
Motor proficiency	Gross motor skills Motor coordination Fine motor skills Total score of motor proficiency	8.69	10.60
		3.15	5.08
		7.34	8.17
		18.94	21.20
Executive function	Inhibition Shift Emotion regulation Emotion regulation index Initiation Planning Organization Monitoring Working memory Metacognition index Total score of motor proficiency	12.07	4.92
		8.69	4.28
		11.65	5.20
		32.36	13.44
		10.13	3.07
		14.82	4.52
		7.34	3.40
		12.00	3.31
		13.55	4.16
		58.01	14.64
		90.53	25.66

**Table 3:** Pearson correlation coefficients between school function and motor skills and executive function

**Variables**	**Items**	**Correlation coefficient**	**P value**
Motor proficiency	Gross motor skills Motor coordination Fine motor skills Total score of motor proficiency	0.44	0.001^***^
		0.47	0.00^***^
		0.62	0.00^***^
		0.56	0.00^***^
Executive function	Inhibition Shift Emotion regulation Emotion regulation index Initiation Planning Organization Monitor Working memory Metacognition index Total score of motor proficiency	-0.06	0.32
		-0.25	0.03^*^
		-0.03	0.40
		-0.11	0.20
		-0.19	0.08
		-0.14	0.15
		0.02	0.43
		-0.29	0.01^*^
		-0.20	0.07
		-0.21	0.06
		-0.18	0.09


***P(v)  0.001

** P(v)  0.01

* P(v)  0.05

## Limitations

One of the limitations of the present study was the lack of access to the same number of students in each grade.

## Suggestions

Considering the importance of participating in school activities, which is a great concern of the parents of children with ASD, it is suggested that future studies examine the role of other factors such as social performance, linguistic skills, etc. in determining the extent of participation of these children in various school activities. Also, according to the results of this study, it is suggested that the effects of perceptual-motor interventions and cognitive rehabilitation be assessed on these children’s school participation rates.

## In Conclusion

The findings of this research showed the direct relationship of autism children’s participation in school activities with their motor proficiency and performance in the shift (i.e., behavioral regulation index) and monitoring (i.e., the metacognitive index) aspects of executive function. In assessing the relationship of school participation with the two factors of motor proficiency and executive function, motor proficiency had a stronger correlation with participation in school activities. It is suggested that perceptual-motor interventions and cognitive rehabilitation programs (with a focus on the shift and monitor aspects) be implemented to increase the participation of children with ASD in school activities.
